# Direct infrared spectroscopy for the size-independent identification and quantification of respirable particles relative mass in mine dusts

**DOI:** 10.1007/s00216-020-02565-0

**Published:** 2020-04-14

**Authors:** Robert Stach, Teresa Barone, Emanuele Cauda, Patrick Krebs, Bobby Pejcic, Sven Daboss, Boris Mizaikoff

**Affiliations:** 1 Institute of Analytical and Bioanalytical Chemistry, Ulm University, Albert-Einstein-Allee 11, 89081 Ulm, Germany; 2 Pittsburgh Mining Research Division, Centers for Disease Control and Prevention (CDC), National Institute for Occupational Safety and Health, Pittsburgh, PA 15236 USA; 3 CSIRO Energy Flagship, 26 Dick Perry Ave, Kensington, WA 6151 Australia

**Keywords:** Infrared spectroscopy, IR, Fourier transform infrared spectroscopy, FTIR, Diffuse reflectance, DRIFTS, Inhalable particles, Alpha quartz, Crystalline silica, Mineral dust, Occupational safety, Mining, Multivariate data analysis, Chemometrics, Partial least squares regression, PLSR

## Abstract

Due to the global need for energy and resources, many workers are involved in underground and surface mining operations where they can be exposed to potentially hazardous crystalline dust particles. Besides commonly known alpha quartz, a variety of other materials may be inhaled when a worker is exposed to airborne dust. To date, the challenge of rapid in-field monitoring, identification, differentiation, and quantification of those particles has not been solved satisfactorily, in part because conventional analytical techniques require laboratory environments, complex method handling, and tedious sample preparation procedures and are in part limited by the effects of particle size. Using a set of the three most abundant minerals in limestone mine dust (i.e., calcite, dolomite, and quartz) and real-world dust samples, we demonstrate that Fourier transform infrared (FTIR) spectroscopy in combination with appropriate multivariate data analysis strategies provides a versatile tool for the identification and quantification of the mineral composition in relative complex matrices. An innovative analytical method with the potential of in-field application for quantifying the relative mass of crystalline particles in mine dust has been developed using transmission and diffuse reflection infrared Fourier transform spectroscopy (DRIFTS) within a unified multivariate model. This proof-of-principle study shows how direct on-site quantification of crystalline particles in ambient air may be accomplished based on a direct-on-filter measurement, after mine dust particles are collected directly onto PVC filters by the worker using body-mounted devices. Without any further sample preparation, these loaded filters may be analyzed via transmission infrared (IR) spectroscopy and/or DRIFTS, and the mineral content is immediately quantified via a partial least squares regression (PLSR) algorithm that enables the combining of the spectral data of both methods into a single robust model. Furthermore, it was also demonstrated that the size regime of dust particles may be classified into groups of hazardous and less hazardous size regimes. Thus, this technique may provide additional essential information for controlling air quality in surface and underground mining operations.

**Figure:**
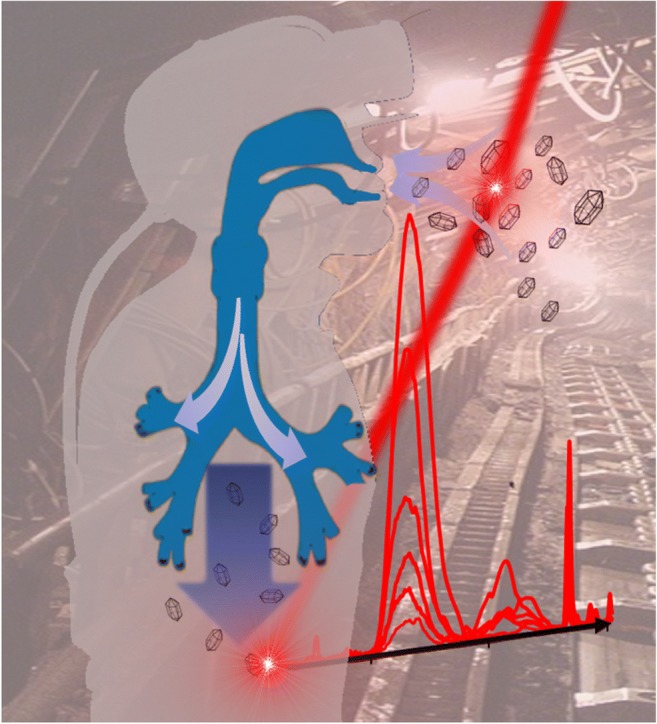
Graphical Abstract

Graphical Abstract

## Introduction

The exposure of mine workers to crystalline particles in the respirable size regime is a global problem in occupational health and safety at surface and underground mining operations [1–5]. For inhaled particles, from which 50% can penetrate the larynx (i.e., thoracic particles, 10 μm) and especially those size regimes from which 50% of the mass fraction may enter unciliated airways (i.e., respirable particles, 4 μm), in-field online monitoring of the composition and concentration is needed for the assessment of hazards and intervention [2, 6]. The focus of recent studies is especially targeted to respirable crystalline silica (alpha quartz), which is known to cause a variety of lung diseases including chronic obstructive pulmonary disease (COPD) and silicosis. Alpha quartz was even associated with higher risks of lung cancer and is suspected to be a lung carcinogen [7–9]. For occupational safety and health, the hazardous effects during the exposure of mine workers to dust containing respirable crystalline silica particles was unambiguously proven during past studies [4, 10–12]. A permissible exposure limit (PEL) of 100 μg/m^3^ respirable dust present in metal/non-metal mines was prescribed by the U.S. Mine Safety and Health Administration (MSHA). The National Institute for Occupational Safety and Health (NIOSH) introduced a recommended exposure limit (REL) of 50 μg/m^3^ as a time-weighted average for up to 10 h per day during a week of 40 work hours [2, 13].

Besides the respirable crystalline silica particles, other particles in the respirable size regime are also suspected of causing pulmonary diseases (e.g., dolomite, calcite, or aluminosilicates) [14–16]. Thus, concerning occupational safety and health of exposed workers, there is a demand for selective, or more precisely, comprehensive particle monitoring, which includes dust particle identification, differentiation of the mineral composition, quantification, and size of the particles.

To date, the challenge for rapid in-field monitoring of respirable particles has not been solved satisfactorily, in part because conventional analytical methods, including for example X-ray diffraction (XRD), require laboratory environments, complex method handling, and tedious sample preparation procedures. Additionally, the quantification is in part limited by particle size effects as a confounding factor [17, 18].

A commonly adopted standard method for the quantification of crystalline silica is the NIOSH 7500 XRD analysis [2]. At least in part, real-time methods based on light scattering can be used but are restricted to the presence of particles and cannot differentiate the type of dust particles and their composition for quantifying their hazardous potential [19].

Our research teams have demonstrated that Fourier transform infrared (FTIR) spectroscopy provides a versatile—and nowadays portable—tool for the identification and quantification of various minerals in complex matrices. Due to the inherent molecular selectivity for organic as well as inorganic matrix components, IR techniques allow for precisely determining the mineral composition even in most complex sample matrix scenarios [18, 20].

The first successful results on the in-field quantification via the NIOSH direct-on-filter (DoF) method combined with portable FTIR spectrometers have proven to be of comparable quality to the NIOSH 7500 method [21]. In that study, mine dust particles were directly collected onto PVC filters by the respirable samplers [2, 22]. The DoF method combined with the FTIR technique is included in ISO 19087:2018 by the International Organization for Standardization [23]. However, analytical confounders such as variable background and effects of mixed mineral components in the analyzed dust sample matrix have been shown to negatively influence the reliability of the data analysis [1, 22, 24]. Those effects are most effectively addressed by single variable data evaluation strategies, as shown via a direct calibration approach for coal and non-coal mine dust using partial least squares regression (PLSR) [1, 22]. Most recent studies by Cauda et al. [2] confronted the problem of confounding variables with a sector-specific and a mine-specific approach.

In the present study, an alternative analytical method with the potential of in-field application for the quantification of the relative mass of a variety of crystalline particles in the respirable regime has been developed, combining transmission and diffuse reflection infrared Fourier transform spectroscopy (DRIFTS) within a unified multivariate model.

Transmission IR and DRIFTS have individually been tested as a viable method for the quantification of respirable crystalline silica [25]. However, the performance of DRIFTS is highly susceptible to particle size [26, 27]. In the present study, this potential weakness was, in fact, turned into an advantage by utilizing that dependence for deriving particle size as an additional variable besides the total particle mass and mineral composition within a unified multivariate model.

For establishing a proof-of-principle study, a laboratory calibration was established using a set of mixed relevant minerals in limestone mines, i.e., calcite, dolomite, and quartz embedded into a KBr matrix in accordance with XRD and energy-dispersive X-ray spectroscopy (EDX) mapping data of various real-world limestone mine dust samples.

Thereby, the calibration/classification strategy was tailored to the matrix and composition of dust samples occurring in real-world underground mining scenarios, which effectively addresses the issue of matrix-based analytical confounders along with the identification and quantification of other minerals besides alpha quartz. Using experimental design routines, the size of the required calibration dataset was reduced to a minimum number of calibration samples for providing effective calibration strategies. Thereby, the method may be readily adapted via rapid site-specific calibration routines (e.g., for a specific mining scenario) for the present respirable mineral constituents and matrices with minimum efforts [28].

The predictive performance of the calibration model was demonstrated for two different particle size regimes (i.e., inhalable vs. respirable) and compositions using synthetic laboratory mixtures as well as real-word limestone mine dust samples collected from three different mines within the USA and from one deposit in Berlin, Germany.

This approach has the potential for systematic implementation for on-site quantification of the relative mass of different crystalline particles in ambient air when collected directly onto PVC filters by the worker using body-mounted sampling devices. Without further sample preparation, these loaded filters may be directly analyzed via transmission spectroscopy and/or DRIFTS, and the mineral amount is immediately quantified via a PLSR algorithm enabling the combining of the spectral data of both methods into a single robust model. Compared to previous studies, this approach, for the first time, enables integrating matrix effects into the calibration/classification model, classifying the size regime of the particles (i.e., inhalable vs. respirable), and quantifying and identifying the relative mass of a variety of different particles with the benefit of rapidly tailoring the calibration to a specific mining scenario (e.g., the mineral composition of a specific mining site or type). In the future, one may, thus, even envisage using this method for *site fingerprinting*, characterizing individual mining locations via their dust composition.

## Materials and methods

### Instrumentation

FTIR measurements were conducted using a portable Bruker Alpha FTIR spectrometer (Bruker Optik, Ettlingen, Germany) equipped with either DRIFTS or a transmission assembly (Bruker Optik, Ettlingen, Germany). For EDX and SEM measurements, a Quanta 3D FEG FIB-SEM dual-column system (FEI, Eindhoven, Netherlands) was used.

### Particle generation

Powders of pure quartz and dolomite (Wards National, CA, USA) were ground in an agate mortar for about 5 min to obtain large (> 5 μm) particles that approximate the inhalable size convention. The inhalable size convention includes particles that can remain airborne and can be breathed into the nose or mouth. The same particles were further ground for more than 130 min to obtain small (≤ 4 μm) particles that approximate the respirable size convention. The respirable size convention includes particles that penetrate beyond the ciliated region of the respiratory tract [6]. Hereafter, these samples are referred to as inhalable and respirable particles. For calcite, optical grade crystals were crushed and ground for approximately 10 min to obtain a powder; then, the calcite powders were treated like the dolomite and quartz samples.

The powders were analyzed via IR attenuated total reflection (ATR) spectroscopy and transmission IR during the grinding process for characterizing size effects (i.e., signal attenuation) and to ensure a minimum size after the procedure. With decreasing particle size, the IR signal approximates a maximum if the particle sizes approach the respirable regime [17]. In addition, the dimensions of the quartz particles after 5 min and > 130 min of grinding, respectively, were spot-checked via scanning electron microscopy (SEM) to verify the abundance of either respirable or non-respirable particles after mixing the minerals according to an experimental design routine (see below). The mixtures were ground for another 5 min to obtain homogeneous samples.

Likewise, limestone mine dust samples from three different mines located in the USA and a sandstone sample from Germany (Berlin) were used as real-world examples for determining the predictive quality of the established classification model. The limestone mine dust samples for the experiments herein were provided by NIOSH under subcontract no. 200-2017-M-94234 with permission of scientific usage (locations blinded).

These samples were treated (through the grinding process) similar to the calibration sample ensuring the presence of respirable samples free from agglomerates.

### Creation of the sample set

For the creation of a synthetic sample set, the particles were weighed and mixed. For homogenization, the mixtures were shortly ground in an agate mortar. All mixtures contained calcite, dolomite, and quartz at concentrations according to an experimental design (see section “Experimental design and multivariate data analysis”).

### ATR, transmission, and DRIFTS measurements

For ATR measurements, non-diluted samples were pressed directly onto a single-bounce diamond ATR element without further sample preparation. The pressure was kept steady and reproducible using the pressure indicator of the ATR cell after preliminary tests via pressure-indicating paper foil (Fujifilm Prescale^®^). DRIFTS and transmission measurements were performed using diluted samples containing 2 mg of sample mixture (i.e., 1% wt. sample in dry KBr). For both, pellets and DRIFTS powders for the respective amount of sample were weighed and mixed with KBr in an agate mortar via grinding for approximately 5 min to obtain homogeneous mixtures. For transmission studies, from each mixture, 3–4 pellets using 200 mg of powder each were pressed using a Specac KBr press for 3 min at 10 tons pressure. The derived pellets were analyzed against air as background. For DRIFTS, the raw powder was split into portions of 200 mg for direct measurements in an aluminum cup against a gold cup as background. All spectra were recorded at a spectral resolution of 2 cm^−1^ averaging 128 spectral scans. Each mineral mixture was analyzed five times with each technique for statistical significance and to obtain independent results. The data collection was done using the Opus 6.5 software package (Bruker Optik, Ettlingen, Germany).

### Experimental design and multivariate data analysis

For multivariate data analysis, the Eigenvector Toolbox software package (Eigenvector Research, Inc., Manson, WA, USA) was used. The PLSR method was applied for calibration, quantification, and identification of minerals in calibration mixtures and real-world samples. For calibration, a dataset of 9 mineral compositions (Table 1) was made according to an experimental design strategy. As an experimental design algorithm, an extreme vertice algorithm was applied using the R Statistics software package (The R Foundation, Vienna, Austria) combined with the mincalib add-on package. The extreme vertice design is based on a simplex centroid design with constraints. The constraints (Table 1) were set to match real-world samples with known mineralogy (Table 2).

**Table 1:** Target weight percentages for calibration mixtures according to experimental design. Maximum and minimum values are constraints for the experimental design. Artif. D10 is a laboratory-prepared synthetic mixture following the XRD data of the real-world sample (D10)

Mixture	Calcite (% wt.)	Dolomite (% wt.)	Quartz (% wt.)
I	1	90	9
II	9	90	1
III	5	90	5.01
IV	1	59	40
V	1	74.5	24.5
VI	53.5	45.5	1
VII	59	1	40
VIII	98	1	1
IX	75.5	1	20.5
Valid 1	30	40	40
Valid 2	33.6	48.2	18,2
Artif. D10	45.8	15.5	30.6

**Table 2:** XRD reference data for mineralogical samples provided by NIOSH and Free University (FU) Berlin

Component	Component weight percentage (% wt.)			
	D4	D9	D10	Sandstone Berlin (SSB)
Calcite	20	12	42	33
Dolomite	55	74	15	34
Quartz	20	4	37	24
Others	5	0	0	9

Validation was performed via three validation mixtures including the synthetic sample D10, and in addition four real-world samples, all were included in the validation dataset.

### EDX verification of sample purity

To verify the XRD reference values, all minerals (i.e., calibration samples and natural mine dust) were crosschecked via EDX mapping. Especially for the calibration samples, it is essential to check minerals for impurities, as the minerals are derived from natural deposits. Spot measurements were made on powders immobilized at adhesive carbon pads (Plano GmbH, Wetzlar, Germany) with a resolution of 1024 × 800 pixels. Mainly K-α lines of Al, Si, Ca, and Fe were investigated at every pixel of the image to assign elemental compositions to the respective particles. Thus, obtained mapping data were evaluated using the FIJI software package (LOCI, University of Wisconsin-Madison, USA). Due to the abundance of primarily aluminosilicates or iron within the natural dust samples and not listed by XRD, the data was corrected by derived elemental composition using EDX mapping (Table 3).

**Table 3:** EDX reference data for mineralogical samples

Component	Component weight percentage (% wt.)			
	D4	D9	D10	Sandstone Berlin (SSB)
Calcite	22.4	16.6	43	31.1
Dolomite	53.4	68.4	15	37.7
Quartz	9.3	5.8	37	25.6
Others	15.0	8.1	5	5.7

## Results and discussion

**Fig. 1:**
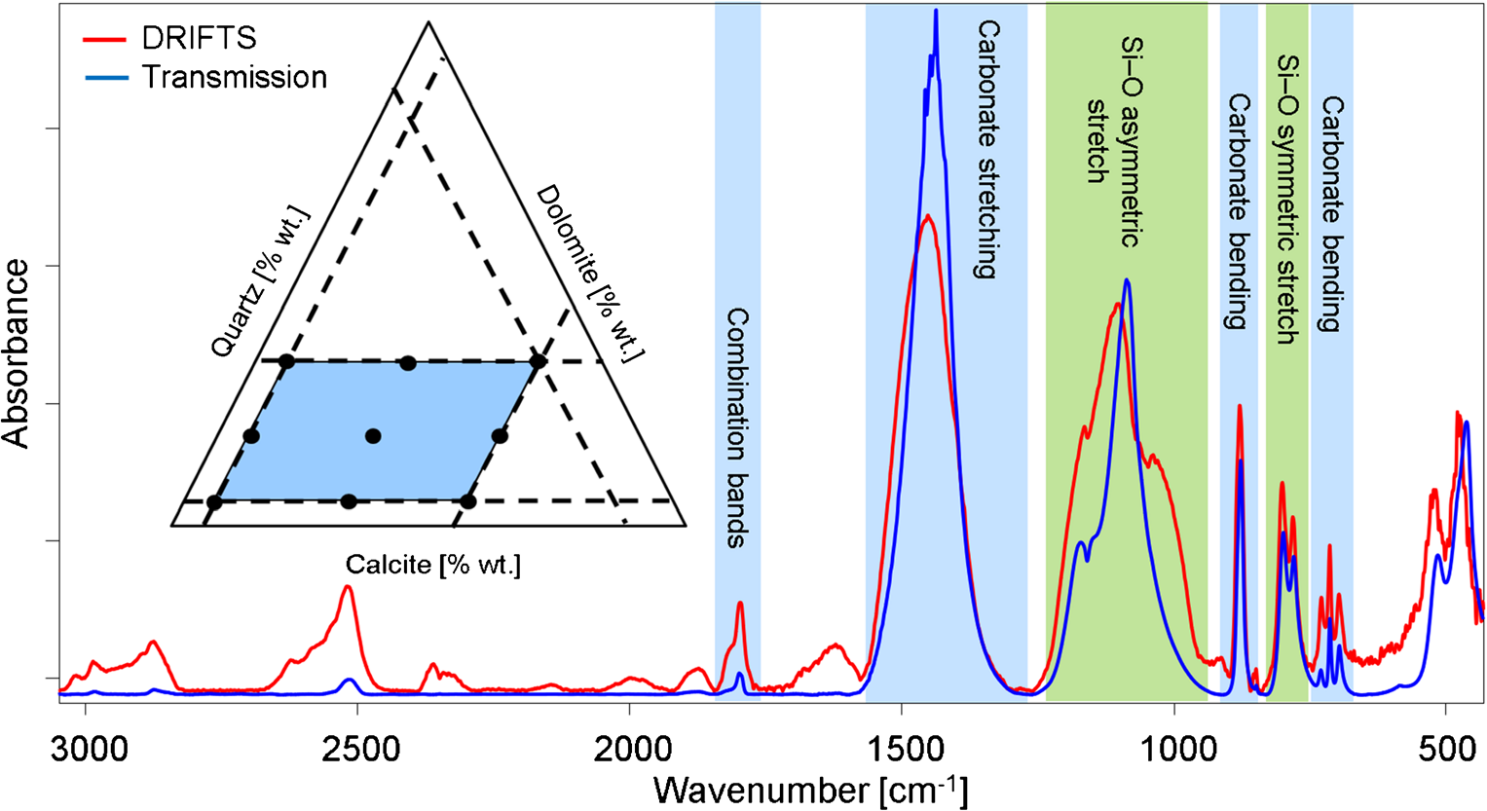
DRIFTS and transmission IR spectra of limestone mixtures and the resulting scheme of the experimental design for quartz, dolomite, and calcite with peak assignments [33]

Using multivariate data analysis strategies, a model that avoids collinearity is essential. For multicomponent calibration samples, this prerequisite leads to a dramatic increase in required calibration mixtures if a fully factorized approach is anticipated. The experimental design algorithm applied in the present study decreases the number of required calibration samples based on a geometric triangular function while avoiding co-linear composition variations. The applied extreme vertice design is based on a simplex centroid with constraints added to the calibration dataset to limit the calibration range of the respective material [28–32]. This application of constraints allows the tailoring of the required calibration mixtures to the specific composition of the real-world samples shown in Table 2. A scheme of the applied ternary design is shown in Fig. 1.

As the most abundant minerals (see Table 2), calcite, dolomite, and quartz were selected as primary target analytes for this proof-of-principle study. The constraints for the experimental design are shown by maxima and minima in Table 1.

The transmission and DRIFTS spectra (Fig. 1) of limestone samples emphasize the need for multivariate analyses in such complex sample matrices. The obtained IR spectra show substantial overlaps of bands (e.g., for silicates and carbonates), and even in these rather simple examples, they exemplify that a simple direct quantification for multiple minerals via peak area integration in such mineral mixtures is limited. Additionally, analytical confounders (i.e., particle size and impurities) add distortions to the already complex IR fingerprint structure. In brief and as commonly known, the PLSR approach applied in the present study reduces the data to the main directions of variance across the entire spectrum by transformation into the space spanned by the partial least squares (PLS) loadings, also called latent variables (LVs) [28–30].

### Quantification of synthetic and real-world mineral particle samples

As transmission IR is the most user-friendly and only method for analyzing DoF filters [21], the prepared calibration samples were measured in KBr along with natural samples from four real-world limestone deposits. The data was preprocessed using baseline correction, normalization, and autoscaling prior to executing PLSR. The concentrations in real-word samples were corrected according to the EDX (Table 3) and crosschecked for confounders to ensure exact masses of the respective minerals and to minimize the effect of impurities on the PLSR predictions.

**Fig. 2:**
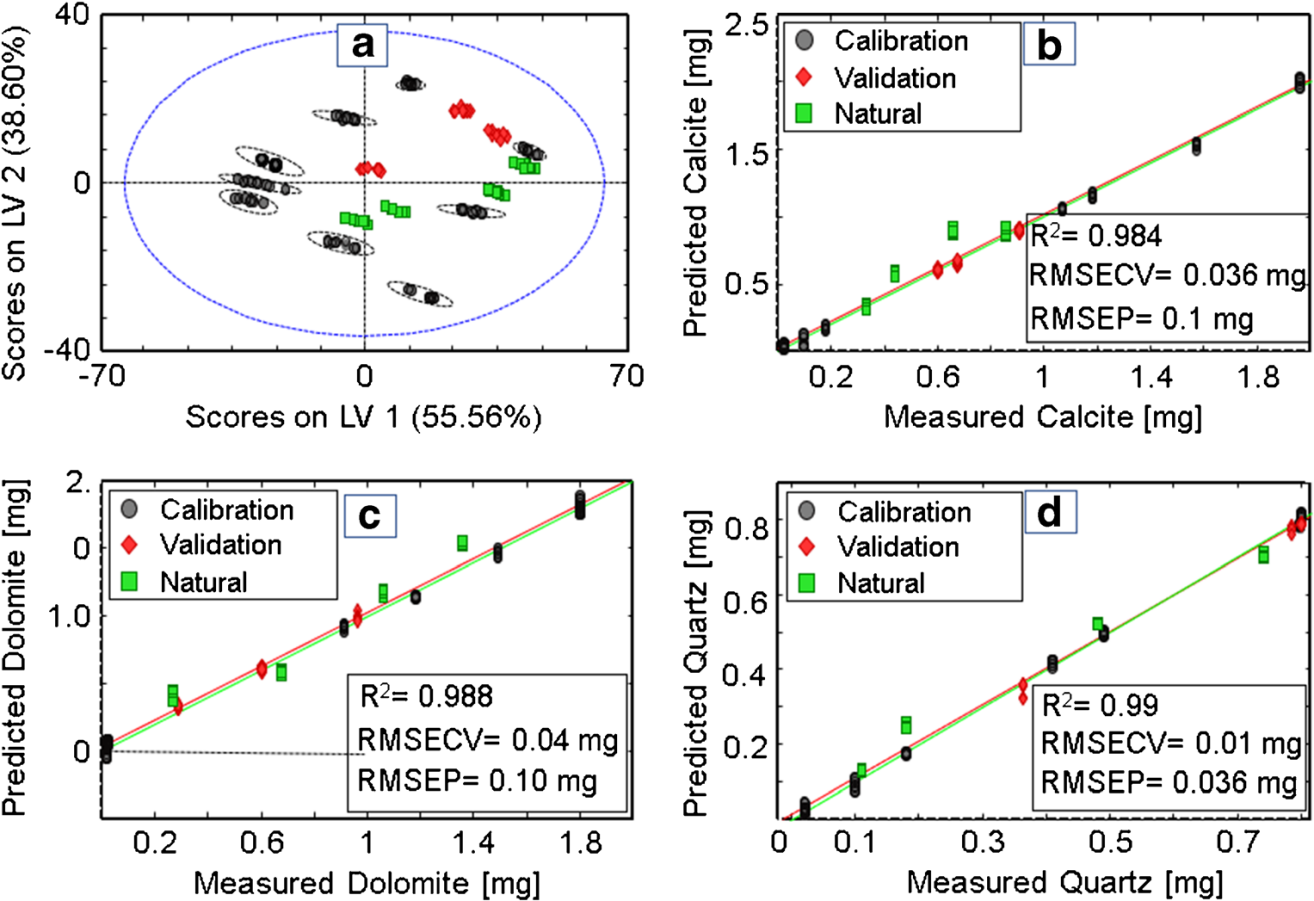
Scores plot (**A**) and PLS regression (**B**–**D**) of respirable calcite, dolomite, and quartz in KBr, including natural limestone samples in the validation set. Black circles represent calibration mixtures. Red rues represent validation mixtures. Green squares represent prediction natural respirable limestone dust samples

As shown in Fig. 2, the scores plot (A) resembles the geometric arrangement of the applied experimental design and can clearly discriminate between all calibration mixtures and the real-word samples indicated by 95% confidence ellipses. This even holds true for mixtures with only minute differences in composition. For the regression, four LVs were selected evaluating the root mean square error of calibration (RMSEC)/root mean square error of cross-validation (RMSECV) versus latent variable number plot. In total, a cumulative variance of 97.45% was covered.

The regression of all three components (Fig. 2B–D) resulted in excellent coefficients of determination of 0.984 and 0.988 for calcite and dolomite, respectively, and 0.99 for quartz. The RMSECV was 0.01 mg to 0.04 mg with the lowest error for quartz. This indicates that each component was calibrated with a pronouncedly low deviation from the reference values, providing excellent correlation. The main error for the predictive quality of the calibration is expressed as the root mean square error of prediction (RMSEP) [29]. All minerals were quantified with satisfactorily small RMSEPs, i.e., 0.1 mg for calcite and dolomite, respectively, and 0.036 mg for alpha quartz. Especially with regard to the fact that some samples only contained small amounts (i.e., < 80 μg) of crystalline silica, the presented predictivity values are excellent. Additionally, the relative error of prediction (REP, %) was calculated as an additional statistic indicator for the quality of the prediction derived from the ratio of RMSEP and the mean calibration value of each component in the validation set (in %). With 15.69% for calcite, 13.68% for dolomite, and 6.90% for quartz, these values are reasonably low considering that 4 out of 7 validation samples are natural mine dust samples. Especially regarding the hazardous alpha quartz, which is much less abundant and thus lower in relative mass compared to the other minerals, the REP% was excellent. However, it has to be noted that the REP% value only indicates the deviation from the mean value of the calibration and does not qualify individual values.

As XRD is the standard method for mineral identification, for completion, the obtained results for the natural samples were compared to XRD measurements performed by NIOSH using DoF filter–sampled respirable particles. Table 4 shows the deviations of results obtained via PLSR versus XRD measurements without EDX correction and crosscheck for confounders.

**Table 4:** Deviation (Δ) results obtained via PLSR versus XRD for the composition of natural samples and lab-made replicates (D10)

Sample	Δ calcite (%)	Δ dolomite (%)	Δ quartz (%)
SSB	36.00 ± 2	− 13.9 ± 2.2	8.8 ± 0.6
D4	27.56 ± 3.6	18.6 ± 2.2	− 37.9 ± 1.7
D9	4.53 ± 5.2	16.1 ± 1.0	61.2 ± 3.5
D10	4.89 ± 3.4	47.5 ± 18.2	− 5.2 ± 1.4
D10 (synthetic)	− 0.82 ± 1.9	12.2 ± 5.8	− 1.4 ± 1.0

The XRD data follows the same trends as the results shown in Fig. 2; however, some show more pronounced deviations. These most evident deviations are found in samples SSB and D4. Overall, the dolomite deviations were most pronounced, while quartz was quantified well except for samples D4 and D9. The latter was anticipated, as only traces of quartz are contained within those samples. However, since XRD is known for a reduced reliability in the investigated small size regime due to intensity loss and line broadening and the fact that XRD measurements of respirable dust are done on DoF filters, this aggravates the quantification due to small sample volumes and filter backgrounds. Hence, the reference values may indeed deviate from the real values in this particle size domain [17, 34, 35]. Additionally, in samples D9 and D10, significant impurities by aluminosilicates were determined, which are not considered via the XRD standard method. Analyzing a lab-made replicate of the XRD quantification of sample D10 prepared by mixing pure minerals (calcite, dolomite, and quartz) shows excellent results with the smallest deviations, which, in turn, substantiates the hypothesis that analytical confounders, filter background, small particle quantities, and impurities severely affected the XRD measurements next to the particle dimensions, which will be investigated in more detail during future studies.

The degree of correlation and predictivity of the presented PLS model already demonstrates that the combination of experimental design and multivariate regression is capable of both rapid identification and reliable prediction of a variety of analytes in multimineral mixtures with minimum simple sample preparation at < 30 s per measurement, which are essential features for in situ assessment of hazards and potential real-time intervention. Once a model is established, any natural sample with matching composition is readily predicted, which is a significant advancement versus current approaches focusing on the quantification of alpha quartz only [2, 21, 25].

### DRIFTS taking particle size into account

For the assessment of hazards and intervention, not only the total mass/number of particles but also the particle size is relevant. However, similar to XRD, IR-based methods show dependencies of the signal strength on the particle dimensions. Contrary to XRD, IR spectroscopy shows an increase in signal strength with decreasing particle size, while XRD conversely is associated with a decreasing signal and broadened lines [17, 35].

Hence, IR-based methods advantageously provide more pronounced analytical signals when approaching smaller, and thus more hazardous, particle diameters. However, the dependence of the spectral features on particle size may lead to higher standard deviations during quantification, especially with increasing inhomogeneity of the particle size distribution. Consequently, an ideal monitoring method for respirable particles should either provide size-independent yet sensitive analytical signals or should be capable of additionally enabling particle size classification. In the present study, we take advantage of the fact that, in general, DRIFTS is more susceptible to the particle size in comparison to transmission IR spectroscopy [36].

**Fig. 3:**
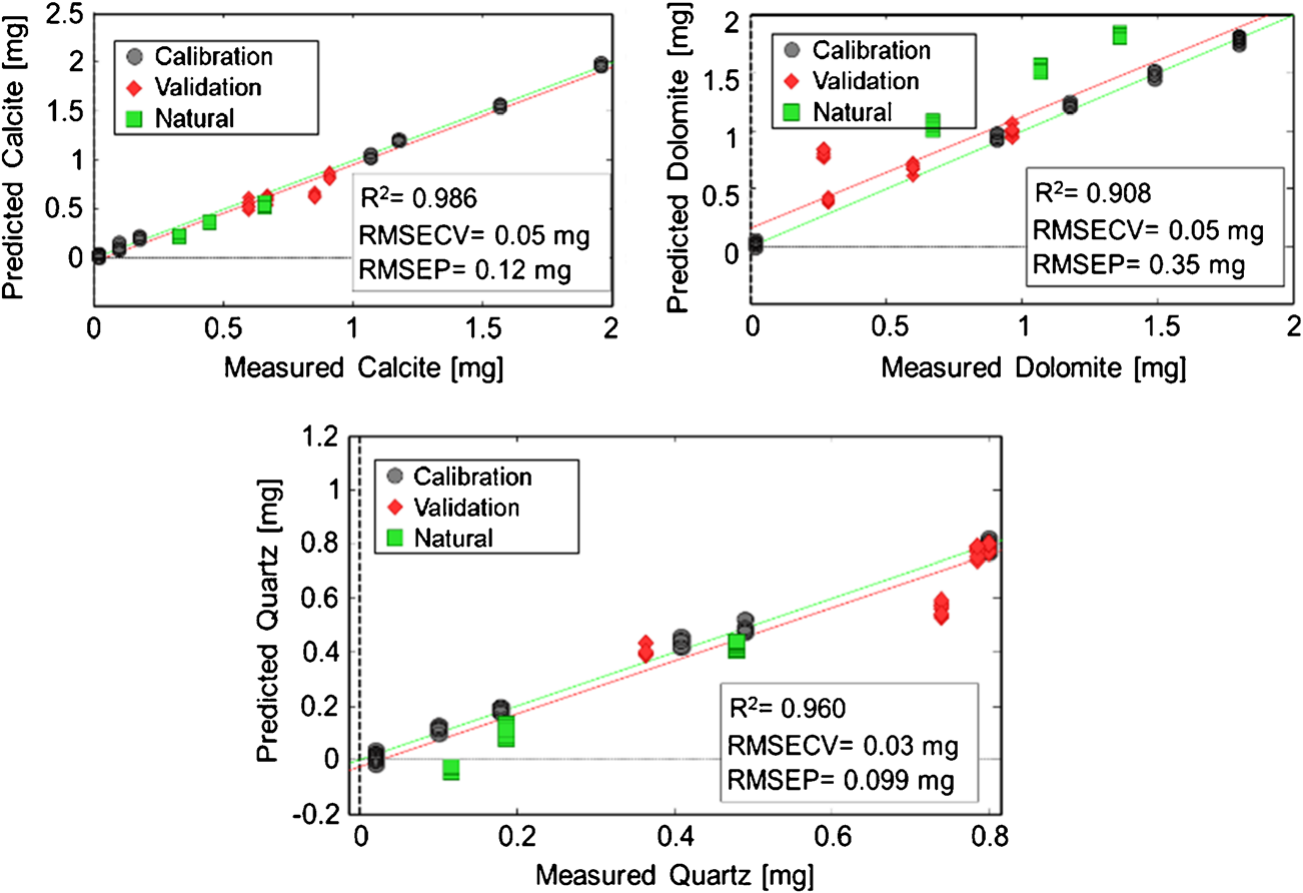
PLS regression of respirable calcite, dolomite, and quartz in KBr analyzed via DRIFTS. Black circles represent calibration mixtures. Red rues represent validation mixtures. Green squares represent prediction of natural respirable samples

In a first step, a multivariate calibration/classification model similar to the IR transmission experiments was established via DRIFTS (Fig. 3).

After baseline correction, normalization, and autoscaling, PLSR was performed, and five LVs were selected covering 80.6% of the total variance. Overall, the DRIFTS model performed well, yet less accurate compared to the transmission IR-based model. The coefficients of determination were 0.986 for calcite, 0.908 for dolomite, and 0.960 for quartz, which are still considered an acceptable correlation. With an RMSEP of 0.12 mg for calcite, 0.35 mg for dolomite, and 0.099 mg for quartz, the three analytes of interest were readily quantified (Fig. 3). While advantageously DRIFTS requires even less sample preparation, the predictive performance of the classification model based on DRIFTS spectra was inferior versus the transmission IR. This is particularly evident in the predicted composition of the natural samples in the validation set, which show higher deviations from the reference values, especially for dolomite. As the natural samples are less uniform in terms of particle size, it is hypothesized that the inferior performance results from particle size effects. Indeed, this is also evident in the latent variable selection, as only 80.6% of the variance is captured despite using five LVs.

### Combining transmission and DRIFTS into a universal particle size–independent model

While it appears that the particle size dependence of DRIFTS is at first glance a negative effect, one may ask the question whether this dependence may, in fact, be utilized for improving the predictive quality of the established calibration/classification models. Ideally, a combined calibration/classification model takes advantage of the accuracy of IR transmission spectroscopy when determining the mineral composition, while at the same time, it utilizes the size-dependent DRIFTS signal for classifying the particle size, as these particle size effects must project into the variances dominating the DRIFTS spectra. Hence, a particle size–dependent parameter was included into the calibration dataset for extracting information on spectral variances in the DRIFTS data caused predominantly by particle size effects. This was enabled by preparing a second sample set using a shorter grinding time, thus achieving an increased average particle size (see also experimental section, i.e., particle generation) to include the spectral behavior of differently sized particles within the calibration dataset.

**Fig. 4:**
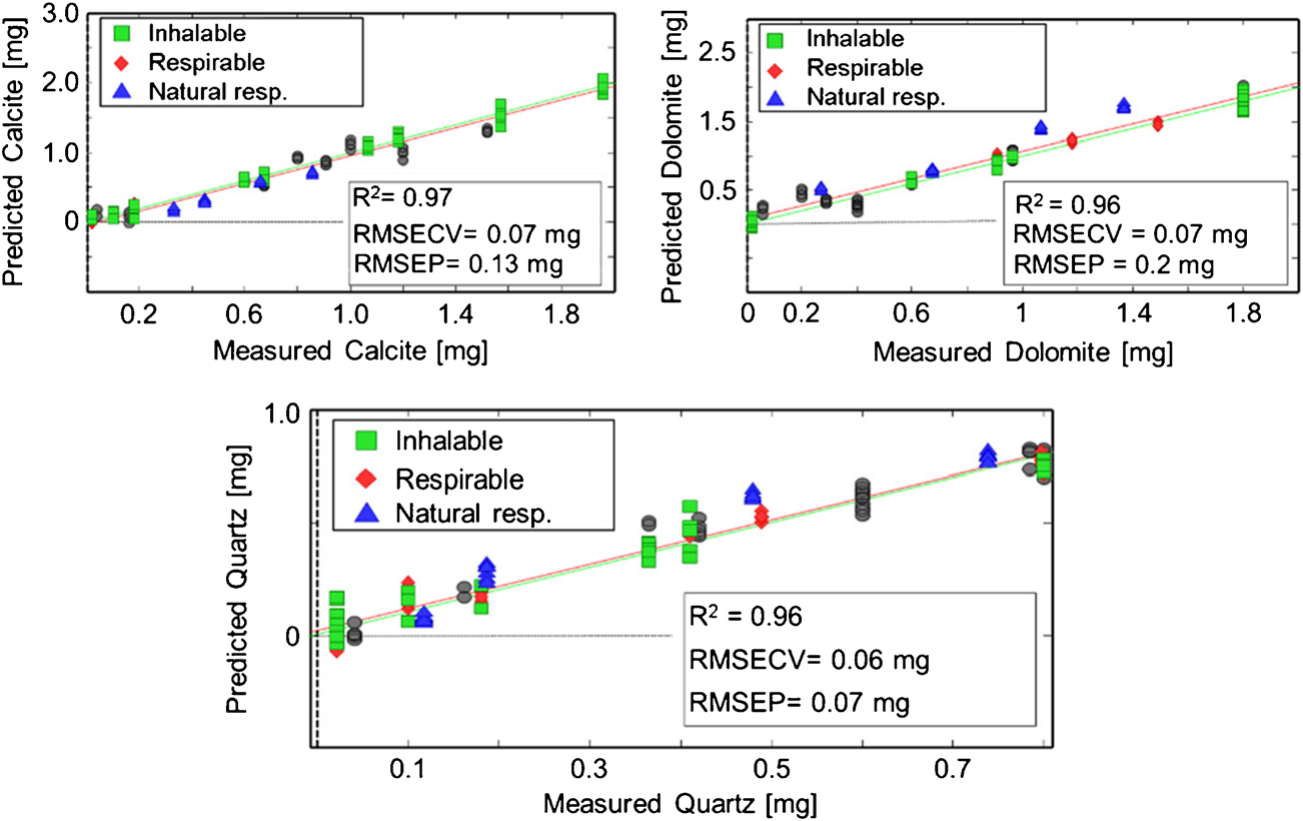
PLSR regression of calcite, dolomite and quartz via a universal calibration model combining DRIFTS and transmission IR data into a single model considering particle size. Green squares represent calibration the dataset based on inhalable-sized particles (< 5 μm) from lab-made mixtures. Red rues represent the calibration dataset based on respirable-sized particles (≤ 4 μm) from lab-made mixtures. Blue triangles represent real-world (natural) respirable samples from Table 3. Black dots represent the validation set

For improving the performance of the overall model, the transmission IR data was included in the PLSR model as well, anticipating superior performance of the resulting universal calibration/classification model. It was expected that the resulting PLSR-based model is capable of characterizing both physical (i.e., particle size) and chemical (i.e., mineral composition and concentration) properties of a sample. The PLSR model based on these combined datasets is shown in Fig. 4.

The sample classes for the dataset set were divided into inhalable (≤ 4 μm) and respirable (> 5 μm) particle size regimes, as well as type, i.e., natural respirable (≤ 4 μm) and laboratory-made (Fig. 4). All data were again baseline-corrected and autoscaled, as spectral baseline drifts resulting from changing ambient conditions in real-world scenarios have to be considered. By evaluating the RMSEC/RMSECV versus the number of latent variables plot, five LVs were selected for covering 93% of the total variance in the combined spectral data block of DRIFTS and transmission spectra, and 98.63% in the concentration data block. The calibration set shows a coefficient of determination of 0.96 for dolomite and quartz and 0.97 for calcite. A superior RMSECV of 0.06 mg quartz and 0.07 mg for for calcite and dolomite was achieved, providing excellent correlation between the experimentally determined and the predicted values.

Each analyte could be predicted with satisfactorily low RMSEPs, i.e., 0.13 mg for calcite, 0.2 mg for dolomite, and 0.07 mg for quartz. Hence, the predictive quality of the model was in between the models using only transmission IR or DRIFT data, however, avoiding detrimental spectral impact of the particle size as an analytical confounder. In particular, the natural samples could be quantified with adequate precision for fast in-field monitoring approaches. The REP% values show the same behavior with 18.2% for calcite, 16.7% for dolomite, and 14.4% for quartz, which are excellent given the deviation from the mean value of the validation samples and the large number of natural samples in the validation block.

**Fig. 5:**
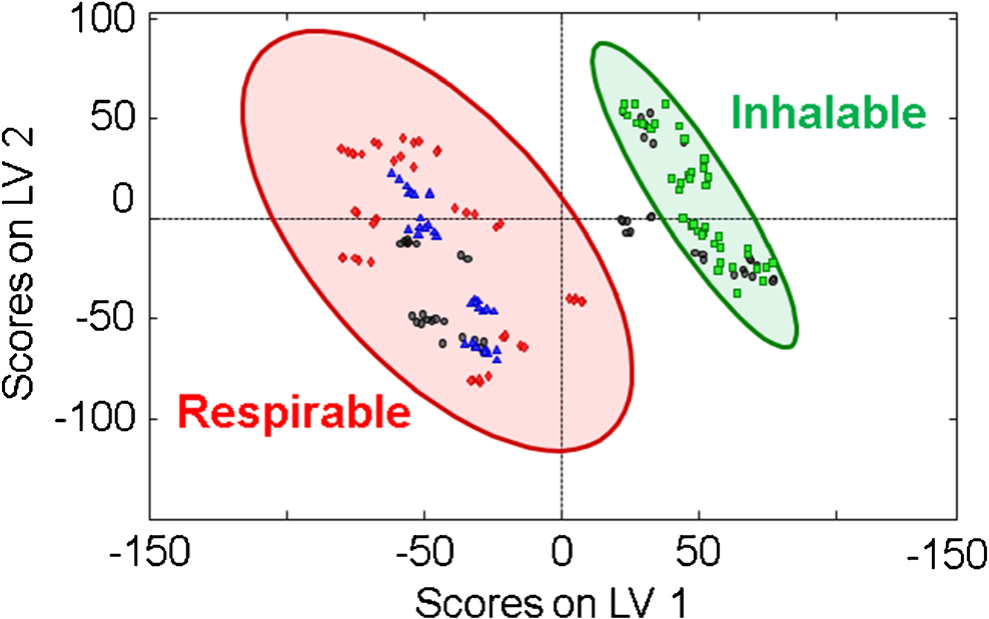
Scores plot based of the combined PLSR model from Fig. 4. Ninety-five percent confidence ellipses illustrate an unambiguous separation between respirable lab-made (red) and respirable real-world (natural) limestone samples (blue) and inhalable-sized (green) particles

In contrast to the PLSR quantifications shown previously based on either IR transmission or DRIFTS data, utilizing both datasets in a combined model enabled the right balance between a sufficiently reduced impact of particle size for accurately determining the composition and capitalizing on the remaining influence of the particle size, especially on the DRIFTS spectra for an inherent particle size discrimination. The scores plot in Fig. 5 illustrates that the two datasets obtained for different particle sizes are clearly discriminated by 95% confidence ellipses based on two latent variables. Most importantly, real-world (natural) samples from limestone mines were unambiguously classified as respirable particles. Hence, the combination of two types of IR spectral data into a universal multivariate model was proven to be capable of quantifying alpha quartz along with other potentially hazardous particles within a limestone matrix, while simultaneously providing a particle size classification into the two relevant two size regimes (i.e., inhalable vs. respirable) to estimate the severity of the exposure scenario.

## Conclusions

In this study, it was demonstrated that direct transmission IR and diffuse reflectance IR spectroscopy combined with a multivariate PLSR calibration/classification model provides a reliable, portable, and field-deployable monitoring platform for determining the relative mass of different mine dust particles and simultaneously give information on the dust composition. Additionally, relevant size regimes may be classified (i.e., inhalable vs. respirable) to rapidly provide information on the hazardousness of an exposure. If samples are collected via PVC filters directly by the worker using body-mounted devices, a minimum of sample preparation is required. Using experimental design algorithms, a strategy for minimizing the number of calibration samples ensuring rapid adaptation to any kind of measurement scenario (e.g., coal mines and quarries), three potentially hazardous analytes—calcite, dolomite, and alpha quartz—within complex synthetic and real-world matrices were quantified with high precision, which expands upon current state-of-the-art techniques determining only alpha quartz.

The combination of data provided by transmission IR and DRIFTS into a universal calibration/calibration model has demonstrated that negative effects on the predictive quality by analytical confounders such as particle size effects may not only be mitigated but may indeed be used to additionally classify particle size without requiring any additional measurements. It is anticipated that a streamlined combination of this strategy with the DoF technique may render this approach the currently most suitable method for rapid on-site assessment of exposure scenarios, justifying potential intervention in underground and surface mining scenarios.
